# Early Follow-up of Parents by a Specialized Cleft Nurse After the Birth of an Infant with Cleft lip and/or Palate

**DOI:** 10.1177/10556656231171750

**Published:** 2023-05-07

**Authors:** Nina Ellefsen Lindberg, Nina Margrete Kynø, Kristin Billaud Feragen, Are Hugo Pripp, Kim Alexander Tønseth

**Affiliations:** 1 Department of Plastic and Reconstructive Surgery, 155272 Oslo University Hospital, Oslo, Norway; 2 Institute of Clinical Medicine, Faculty of Medicine, 6305 University of Oslo, Oslo, Norway; 3 Department of Nursing and Health Promotion, Acute and Critical illness, Faculty of Health Sciences, 60499 Oslo Metropolitan University, Oslo, Norway; 4 Department of Neonatal Intensive Care, Division of Paediatric and Adolescent Medicine, 155272 Oslo University Hospital, Oslo, Norway; 5 Centre for Rare Disorders, Rikshospitalet, 155272 Oslo University Hospital, Oslo, Norway; 6 Oslo Centre of Biostatistics and Epidemiology, Research Support Services, 155272 Oslo University Hospital, Oslo, Norway; 7 Faculty of Health Sciences, 60499 Oslo Metropolitan University, Oslo, Norway

**Keywords:** cleft palate, counseling, feeding, nursing, parental perception, psychosocial adjustment, social support, maternal factors

## Abstract

**Objective:**

To document the impact of early follow-up by specialized cleft nurses (SCNs) to families of infants with cleft lip and/or cleft palate (CL/P).

**Design:**

Prospective inclusion of a control group, which received standard care alone, followed by an intervention group, which in addition received early SCN follow-up.

**Setting:**

The cleft lip and palate team at a University hospital.

**Participants:**

70 families (69 mothers and 57 fathers); control group (n = 38); intervention group (n = 32).

**Intervention:**

SCNs offered a consultation at the maternity ward and follow-ups by phone or face-to-face at one, three, eight weeks and six months after birth.

**Outcome measures:**

Use of Internet-Questionnaire, Quality of discharge teaching scale (QDTS), Post discharge coping difficulty scale (PDCDS), Response on follow-up by health professionals.

**Results:**

Infants in the intervention group were admitted less frequently to a Neonatal Intensive Care unit (NICU); 21.9% vs 51.4%, *P* = .012. Parents in the intervention group used internet for cleft-related reasons less frequently (74.6% vs 85.9%), *P* = .112 and the mothers benefitted less from cleft-related activity on the internet (*P* = .013). The intervention group reported higher mean score for satisfaction with total cleft care (*P* = .001). There were no significant group differences regarding mean total score for discharge teaching (*P* = .315) and coping difficulties (*P* = .919).

**Conclusion:**

Early follow-up by a SCN with expertise in cleft care is highly valued by parents. Closer cooperation between the cleft team and health professionals at birth hospitals and Child health centers is necessary for optimal follow-up.

## Introduction

Becoming parents to a child with CL/P is challenging in many different ways. They clearly need information and support after birth, yet, many report that their needs are not met.^1-3^ One reason is that non-specialized health professionals (HPs) lack competence in cleft care.^4,5^ Because the postnatal period is critical, WHO (2022) have recommended high quality of care to families in general. They consider positive postnatal experiences – defined as information and care offered to families in a consistent manner from motivated and competent HPs who respect their cultural context^6^ – the recommended endpoint. Because satisfaction with HPs is a key predictor of parental well-being and familial adjustment,^7,8^ HPs must strive to meet parents’ and their infants with CL/P's needs, despite lack of consensus about how that should be done.

An immediate concern for parents of children with CL/P is how to feed and care for the infant.^2,4,9,10^ Different cleft types have different structural and functional consequences that affect feeding; e.g., infants with cleft palate have more eating difficulties because of their inability to seal off the nasal cavity and nasopharynx from the oral cavity.^11^ Parents have also observed that the cleft impacts the infants’ health related to sleep, development, comfort and breathing.^12^

Parents of children with CL/P are likely to face psychosocial challenges,^13,14^ e.g., shock and worry at the time of diagnosis, as well as conflicting emotions of delight and grief.^13,15^ Both parents, particularly mothers, report more stress than those of children without CL/P and compared to normative data for the measure used,^7,16^ but they also report stress-related growth and positive adjustment.^17^ Social support and active use of approach-oriented coping strategies seems to help parents adjust and cope.^17,18^ Mothers have described that their coping process was positively affected by the infant's father and other close family. They also expressed a strong desire to make the best choices regarding feeding, nutrition and care for their infants, which were actions they considered important to be a good mother.^4^ That mothers of children with CL/P may prioritize the infant's needs before their own,^4^ is also reported from the broader literature.^19^

Parents of children with CL/P are highly satisfied with the overall healthcare received after diagnosis and treatment, yet many report lacking information and support.^20-23^ A well-documented challenge is that HPs without expertise in CL/P often are the first the parents meet after receiving the diagnosis or after birth.^4,5^ HPs without disease-specific expertise could mistake CL/P-related feeding difficulties to be caused by other factors and therefore unnecessarily readmit the child to hospital.^24^ Others described that conflicting information led to insecurity and skepticism to HPs’ advice and a need to seek sources outside the hospital.^2,4^ Most families turned to social media to seek knowledge about their child's diagnosis.^25^ Commonly posts focused on seeking information and sharing experiences.^26,27^ Despite the benefits of the use of social media, challenges related to misinformation and inappropriate language were reported.^27^ Additionally, findings indicate that adults in general may find it difficult to understand and evaluate the reliability of health information on the internet.^28^

According to Benner (1995), expertise in nursing is developed by being close to the field of practice over time.^29,30^ Based on this, we believe that nurses or HPs who work in or close to multidisciplinary cleft teams have the best prerequisites for possessing the expertise needed to meet parents’ needs, in their process of adjusting to the consequences of the child's condition. This is in line with previous research that found that professional support can be related to specific persons in multidisciplinary cleft teams and that the contribution of a specialist nurse is highly esteemed.^21^ Organization of cleft care in the United Kingdom (UK) and Denmark (DK) have long traditions of facilitating expertise through clinical nurse specialists (UK) and specially trained health visitors/nurses (DK) to families affected by clefts. They provide information, support and feeding guidance, from the time of diagnosis through the first surgical interventions.^24,31-35^ Parents describe these professionals as mediators between themselves and other specialists in the cleft team. They highly value the continuity, competence and emotional support provided by these professionals.^24,34^ Although, these studies document parental experiences and impacts of early follow-up by clinical nurse specialists, only a few have systematically compared actions of care provided to families after birth of an infant with CL/P. Nevertheless, effects of home visits provided by nurses to mothers and infants^36^ and a feeding intervention^37^ have been documented. More knowledge is needed about how to support parents to adjust positively to the challenges associated with their infant's CL/P.

Our aim with this study was to document the impact of follow-up for six months, provided by specialized cleft nurses (SCNs) to parents with infants with CL/P compared to a control group who received standard care, on the following parameters:

- Admittance to NICU in the first postnatal month
- Parent experience of internet use regarding CL/P
- Parent assessment of discharge information at the maternity ward
- Parents’ coping difficulties after discharge from maternity ward and/or NICU
- Parents’ response with follow-up provided by HPs

## Methods

### Design

In this prospective study^38^ of families who received standard CL/P care for an infant, about half of the total sample were also offered follow-up by a SCN.

### Setting

In Norway, were the study was performed, the prevalence of CL/P is about 2.1 per 1000 live births.^39^ About 120 children are annually referred for surgery^40^ and a prenatal diagnosis is revealed in 70% of mothers of children with cleft lip ± cleft palate.^41^ The country has two multidisciplinary cleft teams; the largest one at the hospital were the study was performed. Plastic surgeons, speech pathologists, orthodontists, psychologists, ENT-doctors as well as nurses are among the team professionals. The nurses, titled cleft nurses, have a bachelor's degree in nursing, no formal competence but clinical experience in cleft care. Both teams cooperate on treatment protocols, research and development, and have documented medical and treatment data on patients born with clefts since the early 1960`s.^42^ Health services provided to the Norwegian population are organized within the healthcare system and regulated by laws and guidelines.^43-45^ For families affected by CL/P, the Specialist health service is responsible for cleft treatment and maternity care. The Primary health service is responsible for municipal Child health centers and health services provided to pregnant women and young children, including those with CL/P. The public health nurses at the Child health centers have no extended role regarding cleft care but provide general follow-up. The cleft nurses provide prenatal guidance when couples are referred to a cleft team. The infants with CL/P undergo surgical interventions 1-3 times during the first year, depending on cleft type and individual needs. Cleft lip surgery is done at the age of 3-5 months, often in two sessions if the cleft is bilateral, and cleft palate surgery at 1 year. The state of Norway covers most healthcare expenses (cleft treatment included), follow-up, travelling costs to hospitals and loss of income.

### Procedure

Parents of infants with CL/P were consecutively asked to participate if they met the following inclusion criteria; able to speak, read and write Norwegian; referred to the cleft team from a birth hospital in time for T_1_ (one month). Additional anomalies, other health challenges or prematurity in the infants were not exclusion criteria. The participants in the control group were included in 2015-2016 and the intervention group in 2016-2017. The control group received written information about the study by post while the intervention group received the information from the staff at the maternity ward. The primary researcher (1) then contacted potential participants and collected signed consent forms. The participants completed questionnaires three times after the birth of the infant; at one month (T1), six months (T2) and 13 months (T3). In this study, outcomes at (T1) and (T2) are documented. The questionnaires were sent by post to each participant, who sent the completed forms to the primary researcher in a pre-invoiced envelope. The primary researcher called the parents before each measure and after 3-4 weeks if a reminder was needed. Participants in the control group received standard care (provided by cleft nurses) while the intervention group received the intervention in addition to standard care. Five cleft nurses, with at least two and up to 30 years of experience with families affected by clefts, underwent a 3-day course and carried out the intervention. These cleft nurses, titled Spesialized Cleft Nurses (SCNs), documented the follow-up provided on a checklist. Data collection was completed by the end of 2018.

### Participants

A total of 36 families with infants with CL/P were eligible for the intervention group and 46 families were eligible for the control group. Two families in each group declined to participate while two families in the control group withdrew and four dropped out before T1. Two families in the intervention group dropped out before T1. Questionnaire response rates for the intervention vs the control group were: at T1, 31 vs 38 mothers and 31 vs 29 fathers; at T2, 28 vs 35 mothers and 26 vs 27 fathers. Some participants only responded at one measure point.

### Standard Care

A cleft nurse contacted the mothers by phone within 2-4 weeks after referral from the birth hospital, unless parents or HPs had made an earlier request. The cleft nurse provided information regarding care, feeding and treatment and was also available for two hours on a contact phone once a week. The families attended a one-day course 4-10 weeks after birth and before the first surgical intervention with other families with infants with a cleft. The course – run by the cleft team – consists of an examination of the infant by a cleft surgeon and information and guidance provided by the other team professionals, included cleft nurses and a user representative.

#### The intervention

The intervention was provided in addition to the standard care described below.

A SCN visited the family at the maternity ward within 36 h after referral from the birth hospital. The SCN provided information about the cleft, treatment and feeding (orally and in writing) to both parents and available staff at the ward. The specialized bottles Medela Haberman® bottle and Grieg Easyfeed® bottle were demonstrated and provided for free. The SCN contacted the public health nurse at the Child health center to inform about the infant and sent written information about CL/P. She made three attempts to reach the public health nurse. The parents were then followed up on the phone – at one, three and eight weeks and six months after birth – to discuss potential questions and concerns, or to refer the infant to a public health nurse or arrange a visit at the hospital. The SCN was available on the phone during daytime for the parents, HPs at the maternity ward and staff at the Child health center. The SCNs completed a checklist for each family.

## Measures

**Demographic and background data** – a study-specific questionnaire – provided data on parental gender, age, marital status, educational level and employment status as well as data about their infant; gender, cleft type, birth weight, gestational age at birth, birthplace and birth method, prenatal diagnosis, length of stay at the maternity ward after birth, any admission to Neonatal Intensive Care Unit (NICU) as well as follow-up by HPs.

The questionnaire **Use of internet** –developed for the study – consisted of seven items on the participant's use of cleft-related activity on the internet. The participants were asked if they used the internet to collect information about CL/P; if yes, which sources they used (eg., Facebook, Google) and which topics (diagnosis, treatment, feeding etc) they searched for. They were also asked to score the experienced usefulness of the activity on an 11-point Likert scale (0 = not at all, 10 = very much).

**Quality of discharge teaching scale** (QDTS) consists of 18 items that measure parents’ perception of the quality of discharge teaching, provided by nurses throughout the hospitalization.^46^ The Content subscale consists of six paired items reflecting the amount of *content needed* and *content received* in preparation for discharge. Care for the child, medical needs and treatment, who and when to call for assistance, parents’ emotions as well as information to family members are topics that parents are asked to rate. The Delivery subscale −12 items – concerns skill of the nurses as educators about discharge teaching. It includes items about listening, ability to express sensitivity to personal beliefs and values, promoting confidence in ability to care for the child and providing consistent information. The respondents rate on a scale from “0” (not at all) to “10” (extremely, completely or a great deal).^46^ Cronbach's alpha reliability coefficient for a parent sample was 0.88 for the total scale, 0.78 and 0.88 for content received and delivery scales and 0.70 for content needed scale.^46^

**Post Discharge Coping Difficulty Scale** (PDCDS) – 11 items – concerns parents’ coping difficulties after discharge from hospital after giving birth; e.g., difficulties with stress, recovery, family self-care and management, support, confidence, and child's adjustment.^46^ The scale goes from “0” (not at all) to “10” (extremely, completely or a great deal). Higher total scores mean more coping difficulties. The first five items include respondents’ clarification of the meaning of their quantitative response, and will not be presented in this article. Cronbach's alpha reliability coefficient for a parent sample was 0.84.^46^ PDCDS and QDTS were translated into the country's language by the researchers in line with “The guideline for process and cultural adaption of instruments” provided by WHO^47^ and includes back-translation.^48^

**Response to the follow-up by HPs –** an 18-item questionnaire – was developed for the study in collaboration with the Cleft Lip and Palate Association in the country. The questionnaire measures parents’ perception of aspects of cleft-related care provided by SCNs, cleft team and local HPs (public health nurses, general practitioners or staff at local hospitals).

Six items reflect the control group's preferences and the intervention group's experiences regarding some elements of HPs contribution; e.g., the parents in the control group were asked to score how they *would prefer* aspects of cleft- related care provided by the cleft team and HPs at the Child health center; the intervention group rated how *they experienced* the same topics. Each item was rated on an 11-point Likert scale (0 = not at all, 10 = very much).

### Ethics

The study was exempt from ethical approval by the Regional Committee for Medical and Health Research Ethics, section South-East C, Norway (IRB 00001870). The Data Protection Office at Oslo University Hospital approved the study (Reference number: 2014/17828). The study were registered it at ClinicalTrials.gov.NCT02415361. The participants received oral and written information and signed a consent form before they were included in the study.

## Statistical Analysis

SPSS version 26 (IBM Corp., Armonk, NY) was used for analyses. Cohen *d*^49^ was used to calculate effect size. Due to expectations of medium/strong effect for both mothers and fathers equivalent to an assumed mean difference between groups of 0.8 relative to the standard deviation, we needed to include 26 mothers and 26 fathers in each group to obtain a 80% statistical power at 5% significance level using an independent samples t-test. To summarize data, we used descriptive statistics of means and standard deviation for the continuous variables and counts and percentages for the categorical variables. The Independent sample t-test was used to analyze group differences for the continuous variables, and the Pearson's Chi-Square test and Fisher's exact tests for categorical variables. A two-sided *P* value of <.05 was considered significant.

## Results

The study population consisted of 70 families; 32 in the intervention group and 38 in the control group (Table 1). The groups were comparable regarding baseline characteristics for the parents; for the infants, those in the intervention group had a lower birth weight (3269 g vs 3594 g, *P* = .026) and a lower number represented with cleft palate ± lip (62.5% vs 71.1%). Infants in the intervention group were admitted to the NICU less often than infants in the control group (21.9% vs 51.4% *P* = .012).

**Table 1.** Demographic and Clinical Characteristics; Numbers + % Unless Otherwise is Stated.

	Intervention group (N = 32)		Control group (N = 38)		
	Mother	Father	Mother	Father	
	*(n = 32-31)	*(n = 32-30)	*(n = 38-36)	*(n = 29-28)	*P* value
Parental age (mean ± SD)	31.6 (5.4)	33.6 (5.7)	32.4 (5.4)	33.2 (5.5)	.609
Civil status: married/cohabitant	31 (100)	30 (100)	37 (97)	29 (100)	.541
Employment (paid)	27 (87.1)	28 (93.3)	33 (86.8)	28 (96.6)	.772
Education level					.514
Lower/upper secondary school	8 (25.8)	10 (33.3)	8 (21.1)	10 (35.7)	
University level less/more than 4 years	23 (74.2)	20 (66.7)	30 (78.9)	18 (64.3)	
Prenatal diagnosis received	17 (53.1)		17 (44.7)		.484
Prenatal diagnosis, gestational week (mean ± SD)	18.2 (1.3)		19.1 (2.4)		.725
Prenatal follow-up of CLP-team	17 (54.8)		17 (44.7)		.404
Gender of infant					.099
Girl	9 (28.1)		18 (47.4)		
Boy	23 (71.9)		29 (52.6)		
Cleft type					.433
Cleft lip, unilateral/bilateral	12 (37.5)		11 (28.9)		
Cleft palate	8 (25.0)		15 (39.5)		
Cleft lip and palate, unilateral/bilateral	12 (37.5)		12 (31.6)		
Other health issues					.991
RS, additional anomaly, examinations	4 (13.8)		5 (13.9)		
Gestational week, birth	39.0 (2.2)		39.6 (1.4)		.194
Birth weight, gram (mean ± SD)	3269.0 (663.1)		3594.4 (536.5)		.026
Sibling/s	20 (62.5)		21 (55.3)		.540
Method of delivery					.985
Vaginal	27 (84.4)		32 (84.2)		
Caercarian	5 (15.6)		6 (15.8)		
Birth at hospital	32 (100)		38 (100)		1
Days at maternity ward	3.4 (2.1)		3 (1.7)		.323
Admitted to NICU in the first posnatal month	7 (21.9)		19 (51.4)		.012
Days before contact with CLP-team (mean ± SD)	1.5 (1.5)		8.8 (7.9)		.000
Days before contact, Child health center (mean ± SD)	10.5 (7.5)		9.4 (4.0)		.492

Abbreviations: RS, Robin Sequence, NICU, Neonatal Intensive Care Unit.

*Note:* *range of number of data for variables/items in the table.

### Use of Internet

At T1, 122 parents responded to this questionnaire (Table 2).

**Table 2.** Use of Internet; Mean + SD Unless Otherwise is Stated.

	Intervention group (N = 32)		Control group (N = 38)		Between groups		Interventg	Controlg			
	Mother	Father	Mother *(n = 36-32)	Father *(n = 28-26)	p-value		*P* value	*P* value	Intervention group	Control group	
	*(n = 29-28)	*(n = 29-27)			M vs M	F vs F	M vs F	M vs F	*(n = 58-55)	*(n = 64-58)	*P* value
Have you used internet for cleft related activity? (numbers + %)					.089	.786	.539	.441			.112
Yes	21 (72.4)	23 (79.3)	32 (88.9)	23 (82.1)					44 (74.6)	55 (85.9)	
No	8 (27.6)	6 (20.7)	4 (11.1)	5 (17.9)					15 (25.4)	9 (14.1)	
Did you search the following topics? (numbers + %)					.017	.462	.169	.078			.024
Diagnosis and treatment	6 (20.7)	11 (37.9)	6 (18.2)	7 (25.9)					17 (28.8)	13 (21.7)	
Feeding	0 (0)	2 (6.9)	0 (0)	1 (3.7)					2 (3.4)	1 (1.7)	
Diagnosis, treatment, feeding	15 (51.7)	9 (31.0)	26 (78.8)	14 (51.9)					24 (40.7)	40 (66.7)	
Did not search the topics (diagnosis, treatment, feeding)	8 (27.6)	7 (24.1)	1 (3.0)	5 (18.5)					16 (27.1)	6 (10.0)	
To what extent have you benefited from the activity on the internet?	5.1 (3.3)	5.3 (3.3)	7.2 (2.8)	5.5 (2.8)	.013	.788	.865	.026	5.1 (3.3)	6.4 (2.9)	.028
To what extent have you gained new knowledge?	5.5 (3.1)	6 (3.0)	7.6 (2.3)	6.0 (2.7)	.005	.962	.549	.019	5.7 (3.1)	6.9 (2.6)	.031
To what extent have you received support and encouragement?	5.6 (3.5)	5.7 (3.3)	6.7 (2.9)	5.2 (2.6)	.185	.552	.875	.052	5.7 (3.4)	6.0 (2.8)	.587
To what extent have you shared experiences, knowledge and emotions?	4.8 (3.6)	4.8 (3.3)	6.6 (2.9)	5.2 (2.9)	.035	.628	.970	.076	4.7 (3.5)	6.0 (3.0)	.036
To what extent have you been worried about the cleft related activity on internet?	4.0 (3.1)	3.4 (2.6)	5.3 (2.9)	3.0 (2.4)	.083	.654	.447	.002	3.7 (3.0)	4.3 (2.9)	.256

Abbreviation: M, mother, F, father.

*Note:* *range of number of data for variables/items in the table.

The text in the origin questionnaire is in the country's language. The English version is translated for the purpose of this article and has not been through the process of translation and cultural adaption.

The parents in both groups mostly used Google, Facebook, Cleft Lip and Palate Association and national medical sites. The parents in the intervention group appeared to have used internet for cleft-related activity less than parents in the control group (74.6% vs 85.6%), but the difference was not significant (*P* = .112). Yet, the mean item score reported by mothers in the intervention group about their experiences of using internet was significantly lower than that for the mothers in the control group; they reported to search less for diagnosis, feeding and treatment (*P* = .017), benefited less from the activity (*P* = .013), gained less knowledge (*P* = .005) and benefited less from sharing knowledge, experiences and emotions (*P* = .035). Significant differences were also found between mothers and fathers in the control group, but no difference was found between parents in the intervention group (Table 2).

### QDTS

A total of 125 parents completed the questionnaire at T1 (Table 3). There were no significant group differences regarding the mean QDTS total score (*P* = .315) or subscale scores. The mean QDTS total score was 5.7 (SD 1.8) for the intervention group and 5.3 (SD 1.9) for the control group. Subscale items (mean scores), differed significantly between mothers and fathers; for *information was given at a suitable time* (Delivery subscale) mothers in the intervention group had higher scores than those in the control group (*P* = .013), for *information on what you needed about your emotions* (Content needed subscale) mothers had higher scores than fathers in both groups (control group [*P* = .037] and intervention group [*P* = .012]) (Supplemental Table 1). The lowest mean score, 4.2-5.1 of 10, was reported on the item *if they received consistent information* from HPs. The mean scores for content difference were low in both groups: −.06 (SD 3.1) in the control group and .25 (SD 1.9) in the intervention group.

**Table 3.** QDTS and PDCDS; Mean + SD of Total Scores and Subscale Scores.

	Intervention group (N = 32)		Control group (N = 38)		Intervention	Control	
	Mother	Father	Mother	Father	group	group	
	*(n = 30-28)	*(n = 29-28)	*(n = 38-34)	*(n = 28-25)	*(n = 59-56)	*(n = 66-59)	*P* value
QDTS							
Content needed	4.5 (2.0)	4.1 (1.8)	4.2 (2.2)	4.3 (2.3)	4.3 (1.9)	4.2 (2.3)	.948
Content received	4.8 (2.3)	4.3 (2.2)	4.1 (2.4)	4.3 (2.3)	4.5 (2.3)	4.2 (2.3)	.421
Delivery	6.9 (1.8)	6.7 (2.3)	6.5 (2.0)	6.4 (1.8)	6.8 (2.0)	6.5 (1.9)	.309
Total score	5.8 (1.7)	5.5 (2.0)	5.3 (2.0)	5.4 (1.9)	5.7 (1.8)	5.3 (1.9)	.315
Content difference	.33 (2.2)	.16 (1.7)	−.11 (2.6)	.02 (1.9)	.25 (1.9)	−.06 (2.3)	.418
PDCDS							
Total score	2.9 (1.6)	2.5 (1.2)	2.8 (1.4)	2.6 (1.3)	2.4 (1.4)	2.7 (1.3)	.919

Abbreviations: QDTS, Quality of Discharge Teaching Scale.

PDCDS, Post Discharge Coping Difficulties Scale.

*Note:* *range of number of data for variables/items in the table.

### PDCDS

A total of 125 parents completed the questionnaire at T1 (Table 3). There were no significant group differences regarding the mean PDCDS total score that was 2.7 out of 10 for both groups (Table 3), but significant differences were revealed on item scores between mothers and fathers across the groups. The mothers mean item score for *the need of emotional support* where higher than the fathers, respectively 5.3 (SD 2.9) vs 3.0 (SD 2.8) *P* = .003 in the control group and 5.6 (SD 2.8) vs 3.6 (SD 2.8), *P* = .000 in the intervention group. Overall, parents across the groups reported low scores (2-3 out of 10) on the item *how difficult caring for the infant had been*, and high scores (8-9 out of 10) on items regarding their coping ability and how well parents and infants adjusted to being home. The mean score on the item of *How stressful has your life been?* were between 6.6-5.2 out of 10 (Supplemental Table 2).

### Response to Follow-up by HPs

A total of 115 parents completed the questionnaire at T2 (Table 4). The mean item score from the participants in the intervention group was higher than that in the control group regarding satisfaction with the total cleft-related follow-up they received from HPs; 8.4 (SD 1.6) vs 7.2 (SD 2.1), *P* = .001. The mean score was lower regarding time spent to get into contact with HPs 2.0 (1.9) vs 3.3 (SD 3.0), *P* = .002. The mean score reported by mothers and fathers in the intervention group regarding how useful the visit by the SCN at the maternity ward was, was 8.7 (SD 2.2) and 8.9 (SD 1.4) of 10.

**Table 4.** Response to the Follow up by HPs.

	Intervention g N = 32		Control group N = 38				Interv g *(n = 54-48)	Control g *(n = 61-57)	*P* value
	Mother	Father	Mother	Father	Interv g	Control g			
Regarding cleft related follow up:	*(n = 28-27)	*(n = 26-21)	*(n = 35-33)	*(n = 26-24)	M vs F	M vs F			
1a. To what extent has the follow up you received from the SCN been useful?	7.3 (2.4)	7.5 (1.7)			.710				
1b. To what extent has the follow you received from the cleft team been useful?	7.6 (2.5)	8.3 (1.5)	8.1 (2.1)	8.1 (1.6)	.219	.952	7.9 (2.1)	8.1 (1.9)	.679
1c. To what extent has the follow up you received from local HPs been useful?	3.4 (2.7)	3.4 (2.7)	4.2 (3.5)	5.0 (2.9)	.991	.350	3.4 (2.7)	4.5 (3.3)	.043
2a. To what extent has the follow up you received from the SCN comfort you?	7.7 (2.2)	7.5 (2.0)			.807				
2b. To what extent has the follow up you received from the cleft team comfort you?	8.0 (1.9)	8.7 (1.3)	8.5 (1.4)	8.7 (1.4)	.123	.682	8.4 (1.7)	8.6 (1.4)	.407
2c. To what extent has the follow up you received from local HPs comfort you?	3.8 (3.1)	3.8 (2.7)	4.3 (3.1)	5.0 (2.6)	.978	.341	3.8 (2.9)	4.6 (2.9)	.130
3a. To what extent has the SCN answered your questions regarding diagnosis and treatment?	8.0 (2.2)	7.8 (1.8)			.734				
3b. To what extent has the cleft team answered your questions regard diagnosis, treatment?	8.4 (1.9)	8.7 (1.3)	9.0 (1.8)	8.4 (1.8)	.565	.234	8.5 (1.6)	8.7 (1.8)	.495
3c. To what extent has local HPs answered your questions regarding diagnosis and treatment?	2.4 (2.1)	3.0 (2.5)	2.9 (3.0)	4.0 (2.6)	.339	.135	2.7 (2.3)	3.4 (2.8)	.136
4a. To what extent has the follow up from the SCN made you insecure?	0.6 (1.0)	1.4 (1.4)			.024				
4b. To what extent has the follow up from the cleft team made you insecure?	0.9 (1.4)	1.1 (1.4)	1.8 (2.3)	1.7 (1.9)	.632	.780	1.0 (1.4)	1.8 (2.1)	.021
4c. To what extent has the follow up from local HPs made you insecure?	1.0 (1.6)	1.6 (2.4)	2.8 (2.7)	2.2 (1.7)	.319	.351	1.3 (2.0)	2.5 (2.3)	.003
5a. To what extent has follow up by the SCN been useful regarding feeding your child?	4.8 (2.6)	5.0 (2.9)			.813				
5b. To what extent has follow up by the cleft team been useful regarding feeding your child?	5.1 (3.1)	5.4 (3.1)	5.3 (4.0)	5.7 (2.7)	.730	.653	5.2 (3.0)	5.5 (3.5)	.728
5c. To what extent has follow up by local HPs been useful regarding feeding your child?	3.2 (2.6)	3.0 (3.3)	4.0 (3.5)	4.8 (3.2)	.754	.378	3.1 (2.9)	4.4 (3.4)	.036
6.1. To what extent would it be useful with a visit by a SCN at the maternity ward?			7.5 (2.9)	6.9 (3.3)		.501			
6.2. To what extent was it useful that the SCN visited you at the maternity ward?	8.7 (2.2)	8.9 (1.4)			.639				
7.1. To what extent would it be useful if a SCN contacted you at agreed times?			7.4 (3.0)	6.4 (3.4)		.219			
7.2. To what extent was it useful that the SCN contacted you at agreed times?	7.1 (3.0)	7.6 (2.0)			.469				
8.1. To what extent would it be comforting if a SCN was available on the phone?			8.8 (2.3)	8.2 (2.3)		.316			
8.2. To what extent was it comforting that the SCN was available on the phone?	9.3 (1.6)	8.6 (1.6)			.140				
9.1. To what extent would it be useful if a SCN shared knowledge about CLP with the staff at the maternity ward?			8.7 (2.4)	7.3 (3.0)		.047			
9.2. To what extent was it useful that the SCN shared knowledge about CLP with the staff at the maternity ward?	6.3 (3.8)	6.1 (3.6)			.888				
10.1. To what extent would it be useful if a SCN shared knowledge about CLP with the health visitor at the Child health clinic?			8.0 (3.1)	7.8 (2.4)		.764			
10.2. To what extent was it useful that the SCN shared knowledge about CLP with the health visitor at the Child health clinic?	5.6 (3.5)	5.3 (3.5)			.753				
11.1. To what extent would it be useful if a SCN was a member of the cleft team?			8.6 (2.3)	8.4 (1.8)		.658			
11.2. To what extent was it useful that the SCN was a member of the cleft tem?	8.9 (2.2)	8.7 (1.8)			.715				
12. To what extent have you spent time contacting HPs regarding your child's CL/P?	2.5 (2.0)	1.3 (1.4)	3.5 (2.5)	2.9 (2.1)	.023	.328	2.0 (1.9)	3.3 (3.0)	.002
13. To what extent are you satisfied with the overall follow up from HPs involved regarding your child's CL/P?	8.3 (1.7)	8.6 (1.5)	7.1 (2.3)	7.2 (1.8)	.444	.873	8.4 (1.6)	7.2 (2.1)	≤.001

*Note:* 1a, 2a, 3a, 4a, 5a – completed by the intervention group only.

1b + 1c, 2b + 2c, 3b + 3c, 4b + 4c, 5b + 5c, 12, 13 – completed by both groups.

6.1, 7.1, 8.1, 9.1, 10.1, 11.1 – completed by the control group only.

6.2, 7.2, 8.2, 9.2, 10.2, 11. – completed by the intervention group only.

*range of number of data for variables/items in the table. Abbreviation: M, mother, F, father.

The text in the origin questionnaire is in Norwegian. The English version is translated for the purpose of this article and has not been through the process of translation and cultural adaption.

The mean score of reported usefulness of the SCN being available on the phone was 9.3 (SD 1.6) and 8.6 (1.6) of 10. The mean scores from both groups were moderate to low due to all HPs’ contribution regarding feeding the infant. Furthermore, parents in both groups scored lower for the cleft-related contribution provided by the staff at the Child health centers than those from the SCN and the cleft team.

### Follow-Up – SCNs Check List

The SCNs reported that they spent about 60 min on the first visit at the maternity ward. In three cases, the visits were in the family homes, since the families had already been discharged from hospital. The staff at the maternity ward participated in nine of 34 visits, while there was an exchange of knowledge between the staff and the SCN for 5-30 min in the others, except for the ones already discharged. The SCNs achieved contact with 21 out of 34 public health nurses at the Child health centers. Time spent travelling to and from each family varied from one to 12 h.

## Discussion

In this prospective study, we have documented impacts of an intervention provided by SCNs to families with infants with CL/P compared to families receiving standard care only.

The results showed that in the intervention group, the infants were admitted to NICU less often and the mothers reported to benefit less from the cleft-related activity on the internet. Furthermore, parents in the intervention group were more satisfied with the overall cleft- related follow up from HPs. The groups did not appear to differ regarding discharge information and coping difficulties but gender differences between the parents were revealed.

### Admission to NICU in the First Postnatal Month

Although the infants in the control group were admitted to NICU more often than infants in the intervention group, the overall percentage of admittance to NICU for study infants was higher than in the general population in the Norway, where the admission rate for term infants varies up to 16%.^50^ Studies in the field of CL/P show that infants were readmitted to hospital or NICU after birth because of feeding difficulties, breathing problems and lack of weight gain.^2^ The findings in the present study do not specify the medical reasons for admittances to NICU's nor the routines within the hospitals involved. Nevertheless, the lower percentage of admittance to NICU in the intervention group may be a consequence of SCN presence in the maternity ward after birth. In addition to informing and supporting parents, the SCN may increase knowledge about CL/P and comfort the staff at the maternity ward, specifically regarding how to feed and care for the infant. Furthermore, the presence of a SCN may facilitate joint working and help HPs across the treatment line to share knowledge in practice. That is in line with suggestions from non-specialized HPs to increase expert knowledge in CL/P.^20^ On the other hand, the higher percentage of infants referred to NICU in the control group, could also have been influenced by a higher number of infants with cleft palate in the control group, a cleft type that is known to be more frequently associated with additional medical conditions^51^ and feeding difficulties.^11^ The fact that infants with comorbidities were not excluded from the study could also have increased admission rate to NICU. Additionally, gestational week at birth of infants in the intervention group were lower than the control group; 39.0 (SD 2.2) vs 39.6 (SD 1.4), *P* = .194. As a result, some may have been born prematurely, which is a reason for NICU admissions.^50^

### Use of Internet

Our findings show that parents search traditional internet sites and social media for information about CL/P in line with others.^25^ The SCNs contribution may have affected mothers in particular. Because they reported to benefit less from activity on the internet than the mothers in the control group did, the SCNs seems to have met some of their needs for support and information. The mothers lesser need to search the internet for information could also be a consequence of the fathers being more involved in childcare than those in the control group; e.g., both parents were present at the maternity ward and were informed by the SCN. Our findings indicate that both caregivers should be involved in early care, an action also suggested by others.^52^

### Discharge Information at the Maternity Ward

Surprisingly, there were no group differences regarding discharge information at the maternity ward (total score and subscale scores). We expected that presence of a SCN in the ward would lead to higher scores. Moreover, scores from the “Response questionnaire” showed that a visit by a SCN at the maternity ward was an action that both mothers and fathers in the control group would have preferred. It was also an action that the intervention group valued by giving high scores. Although the QDTS was meant to measure the quality of discharge teaching from the nursing staff as a whole,^53^ one may speculate if the participants in the intervention group included the contribution of the SCN in their scoring or not. For the whole study population, the mean Delivery score (measuring nurses educator skills) and mean Content received score (measuring how much information the parents received) were lower compared to QDTS scores in studies of postpartum mothers and parents of hospitalized children.^46,54,55^ On the other hand, the fact that Content difference were small may indicate that nurses over all meet parent's needs. But the low to medium scoring may also indicate that the parents may have low expectations followed by low demands regarding information and support from HPs because they experience that the staff have little competence with clefts and therefore use other sources of information regarding CL/P.^4^ Findings from a survey of women's experiences with maternity care in the country vary between institutions, especially in regard to information and guidance.^56^ Therefore, more knowledge is needed on factors that promotes and inhibits information and support provided by nurses to families affected by clefts.

### Coping Difficulties in the First Postnatal Month

Overall, parents in both groups reported low levels of coping difficulties in the first month after discharge. Though mean scores is higher than presented by others who have used the PDCDS,^54,57^ findings are still within a middle to low range. The parents reported high item scores (7-9 out of 10) regarding their confidence and ability to care for their child`s needs as well as their own and their child`s adjustment to being home. This may indicate that the parents experienced few difficulties in these areas in the first month after birth, despite the potential feeding difficulties that may occur, especially in the control group were more of the infants had a cleft palate.^11^ This may be seen in the connection of parents reporting that they used little time to get in contact with HPs for cleft related questions, a finding from the Response to the follow up - questionnaire. One may also speculate if their coping strategies were approach-oriented, a finding that has been revealed by others.^17^ Despite this, the question, “how stressful has your life been?” reveal scores that may indicate that although their coping ability may be high, this period is experienced as stressful, a finding that correspond with others.^16,58^

### Gender Differences on Emotional Aspects

Subscale items in QDTS and PDCDS (not total scores) showed that mothers and fathers differ regarding emotional aspects; the mothers needed more information on own emotions and more emotional support in general. This may correspond with others who found that mothers needed to speak about their problems more than fathers, that they had lower self-esteem and were more concerned about being negatively judged by others.^59^ On the other hand, fathers have commented lack of support for men in general, and that they would value individual support.^52^ Considering the gender differences that may occur, a family-based approach may be important to enhance family well-being.

### Response to Cleft Related Contribution from HPs (Regarding Feeding)

Findings from this study show that the participants valued the contribution from HPs with cleft expertise above those without, regarding follow-up after birth. Nevertheless, parents in both groups scored the usefulness of feeding guidance as moderate to low from all HPs involved, including the SCN and HPs from the cleft team. This is in contrast to others who highly value the contribution of cleft nurse specialists regarding feeding.^3,34^ This finding may indicate a potential for improvement in feeding guidance, especially for families with infants with feeding difficulties. For those, advice regarding assessment and feeding technique at the maternity ward followed by information on the phone might have been insufficient. Others also claim that feeding-guidance on the phone may be unsuitable and should be done face-to-face.^35,60^ Yet, the fact that the parents themselves took the main responsibility for feeding their infants may place their own effort above the contribution from HPs. More knowledge is needed on parents’ suggestions for feeding guidance, a knowledge that may be captured by investigating parents’ experiences more in depth.

### Inconsistent Information about CL/P from HPs

The low-to-moderate questionnaire scores regarding consistency of CL/P information from HPs involved, are reflected in SCN comments about difficulties in reaching public health nurses at Child health centers despite three attempts. Though there was knowledge sharing between SCNs and the staff at the maternity wards and Child health clinics, it seems to be insufficient to provide the parents with consistent information. Findings from the broader literature showed that transitions from institutions to municipal health services after giving birth in Norway is a weak point.^56^ Taken together, HPs at maternity wards and at Child health centers have few opportunities to gain the experiences needed to develop expertise in CL/P because they meet relatively few families affected by clefts.^29^ This places a responsibility on the multidisciplinary cleft team to provide colleagues in the municipality and maternity wards with knowledge about CL/P. In light of this, it is important to look at factors that promote collaboration between HPs involved to facilitate consistent and holistic health care to families with infants with CL/P.

## Limitations and Strengths

Our study includes perspectives of both parents in the first months after birth regarding the healthcare they received. By conducting an intervention and comparing the results to a control group, new knowledge about the impact of follow up by SCNs was revealed. Most of the parents who met the inclusion criteria gave their consent to participate. This led to a broad contribution of both mothers and fathers that also seemed to be comparable.

Still, a number of limitations must be acknowledged. First, the lack of heterogeneity between the infants in the groups regarding demographic data may have affected the results, e.g., birth weight, cleft type, gestational week at birth. This is a documented methodological challenge in the field of CL/P and craniofacial anomalies because samples do not contain large enough subgroups.^61^ Furthermore, treatment, cleft surgery, complications or other kind of interventions were not documented, but may have influenced the families, because each factor may in itself have an impact on caring for the infant.

The fact that participants in the control group were investigated one year before the intervention group may also have led to contextual differences between the groups. However, we know that there were no major changes during the organization of healthcare in the study period.

Randomized controlled trials (RCTs) are considered the golden standard for intervention studies, but the method was not feasible in this study because of practical issues and the risk of transference of knowledge and routines between the groups.^38^ If a RCT had been carried out, the risk of transference of knowledge would potentially arise between HPs in practice due to the number of HPs involved in care as well as between participants in the groups, e.g., through activity on social media. In addition, it would have been difficult for HPs and participants to accept that some were offered a visit by SCN while others not, within the same timeframe and context. Based on this, we decided to include the control group before start-up and implementation of the intervention. Worth acknowledging is the possible differences between those who elected follow-up by SCN and those who declined to participate in the intervention group. The fact that only two families declined participation may indicate that parents needed the follow-up offered by the SCN and had trust in nurses^62^ and the healthcare system.

A comment should also be made about the use of multiple statistical tests in this study. That is because the more tests that are carried out, the greater probability of a false positive finding and thereby the risk of drawing wrong conclusion.^63^ There are some opportunities to adjust for multiplicity, but no consensus about when, if or how. The Bonferroni correction – a conservative approach – is referred to as the simplest method. It implies to multiply the p-values with the number of outcomes before comparing with a significant level, and thereby greatly reduce the statistical power.^64^ Consequently, it may be too conservative for the present study. Despite these limitations, we chose to keep the significance level at 5% and to report findings on item level. We believe that the findings on item level have clinical relevance, e.g., the differences revealed between mothers and fathers regarding internet experiences and emotional aspects. These findings add new knowledge to practice and provides inspiration for further research but should be used with caution due to the limitations mentioned above.

Another limitation is that the study measures developed for the purpose of the study have not been checked for external validity. Nevertheless, by developing the Response questionnaire in cooperation with the user organization, we strengthen the ability to measure the real situation, i.e., the preferences and satisfaction of the healthcare the participants received. This process enabled user participation that is valuable and should be central to research.^61,65^ Thus said, many authors of review articles do comment on the lack of consensus and the large amount of measures used in the field of CL/P.^61,66,67^ Suggested research includes development of key constructs and relevant measures and the avoidance of study-specific developed measures if possible.^61^

Finally, the study has been conducted within cultural and contextual frames of a high-income country and a healthcare system, which covers most expenses of healthcare provided to families affected by CL/P. Although the study is relevant, the findings may not be generalizable to countries that organize health care differently or have other health care challenges.

## Summary

Our study provides important insights into parents’ views, preferences and experiences, with an emphasis on the follow-up they received from HPs in the first 6 months after birth.

Follow-up by a SCN facilitates expertise in cleft care, which is highly valued by the parents. To achieve consistent and holistic care, closer cooperation is needed between cleft teams and HPs at birth hospitals and Child health centers. Cleft teams with the expert knowledge in the field have therefore a central responsibility for transference of cleft-specific knowledge. More use of digital platforms may provide opportunities for knowledge sharing, particularly in a country, with long distances between those involved, and high access to internet-based support. More knowledge is needed on the impact of follow-up by SCNs and other HPs on parental stress, infant feeding and well-being.

## Supplemental Material

sj-xlsx-1-cpc-10.1177_10556656231171750 - Supplemental material for Early Follow-up of Parents by a Specialized Cleft Nurse After the Birth of an Infant with Cleft lip and/or Palate Supplemental material, sj-xlsx-1-cpc-10.1177_10556656231171750 for Early Follow-up of Parents by a Specialized Cleft Nurse After the Birth of an Infant with Cleft lip and/or Palate by Nina Ellefsen Lindberg, Nina Margrete Kynø, Kristin Billaud Feragen, Are Hugo Pripp and Kim Alexander Tønseth in The Cleft Palate Craniofacial Journal

sj-xlsx-2-cpc-10.1177_10556656231171750 - Supplemental material for Early Follow-up of Parents by a Specialized Cleft Nurse After the Birth of an Infant with Cleft lip and/or Palate Supplemental material, sj-xlsx-2-cpc-10.1177_10556656231171750 for Early Follow-up of Parents by a Specialized Cleft Nurse After the Birth of an Infant with Cleft lip and/or Palate by Nina Ellefsen Lindberg, Nina Margrete Kynø, Kristin Billaud Feragen, Are Hugo Pripp and Kim Alexander Tønseth in The Cleft Palate Craniofacial Journal

sj-doc-3-cpc-10.1177_10556656231171750 - Supplemental material for Early Follow-up of Parents by a Specialized Cleft Nurse After the Birth of an Infant with Cleft lip and/or Palate Supplemental material, sj-doc-3-cpc-10.1177_10556656231171750 for Early Follow-up of Parents by a Specialized Cleft Nurse After the Birth of an Infant with Cleft lip and/or Palate by Nina Ellefsen Lindberg, Nina Margrete Kynø, Kristin Billaud Feragen, Are Hugo Pripp and Kim Alexander Tønseth in The Cleft Palate Craniofacial Journal
